# Incidence of Otitis Media in a Contemporary Danish National Birth Cohort

**DOI:** 10.1371/journal.pone.0111732

**Published:** 2014-12-29

**Authors:** Tanja Todberg, Anders Koch, Mikael Andersson, Sjurdur F. Olsen, Jørgen Lous, Preben Homøe

**Affiliations:** 1 Department of Otolaryngology, Head & Neck Surgery and Audiology, Rigshospitalet, University Hospital of Copenhagen, Denmark; 2 Centre for Fetal Programming, Department of Epidemiology Research, Statens Serum Institut, Copenhagen, Denmark; 3 Research Unit for General Practice Institute of Public Health, University of Southern Denmark; 4 Department of Otorhinolaryngology and Maxillofacial Surgery, Køge University Hospital, Denmark; CUNY, United States of America

## Abstract

**Objectives:**

In recent years welfare in Denmark has increased which might be expected to reduce otitis media (OM) incidence. We examined the age-specific incidence of OM in a nation-wide cohort of children aged 0–7 years born in 1996–2003 (Danish National Birth Cohort, DNBC). Only selection was ability to understand and speak Danish.

**Methods:**

Information of OM and ventilation tubes (VT) was collected through three maternal interviews at 6-month, 18-month and 7-years of age and based on this age-specific and cumulative incidence of OM was calculated. As different numbers of the total population answered the different interviews, the calculations are done with different denominators. The information in DNBC was validated against two population based registries containing information of VT insertions.

**Results:**

Cumulative incidence of OM at 7 years was 60.6% (31,982/52,755). For children with OM, 16.2% (7143/44194) had their first OM episodes between 0–6 months of age, 44.3% (19579/44194) between 7–18 months, and 39.5% (17472/44194) between 19 months and 7 years. Four or more OM episodes before 7 years were reported by 39.5% (12620/31982) and by 64.0% (2482/3881) of those who had their OM debut between 0–6 months; by 48.2% (4998/10378) with debut between 7–18 months; and by 28.7% (4996/17344) with debut between 19 months and 7 years. These figures are essentially unchanged from earlier figures from Denmark. VT insertion at least once was reported by 26,1% in the 7-year interview. Assuming recordings in the Danish National Patient Registry to be gold standard, maternal self-reportings in DNBC of insertion of VT showed high sensitivity (96.4%), specificity (98.2%), and positive (94.8%) and negative predictive values (98.8%).

**Conclusion:**

OM affects nearly 2/3 of preschool children in Denmark despite reduction in known OM risk factors.

## Introduction

Otitis media (OM) is among the most common infections in childhood [1]. OM comprises acute OM (AOM), OM with effusion (OME), and chronic suppurative OM (CSOM). AOM causes fever, ear pain and discomfort. OME often persists for months and is associated with temporary hearing deficiency [2], which can lead to a delay in learning and language development [3]. The definition of CSOM is not uniform. The WHO define CSOM as more than 14 days of drainage while specialist in otorhinolaryngology often demands 3 months of drainage. However, the more and longer epidodes of CSOM the higher the risk of chronic affection of the middle ear ossicles, permanent tympanic membrane perforation and hearing loss [3].

Worldwide smaller descriptive studies 20–30 years ago showed that OM in Western countries like Denmark was frequent with incidences of 41% and 75% by the age of 3½ and 9 years [4], [5]. However, recently improvements in a number of socio-economic factors (e.g. prolonged maternity leave, less household crowding, less poverty, reduced smoking, introduction of childhood vaccines) have led to improved health. These factors are associated with OM and are expected to reduce the incidence of OM. In contrast, use of day-care centres, a well-known risk factor for OM, has increased markedly. In Denmark there has been no major changes in bacterial resistence patterns in OM associated bacteria and also there has been no change in the restrictive policy concerning antibiotic treatment for OM during these years. The net effect on the incidence of OM is unknown.

Recently, a number of birth cohort studies of varying sizes in different countries have addressed OM [1], [6]–[10]. However, societal factors have changed and it is now relevant to estimate the frequency of OM today. In 1996 the Danish National Birth Cohort (DNBC) was established, covering >100,000 unselected Danish pregnant women and their coming childrens pre- and postpartum living and health conditions [11]. The DNBC is a Danish nationwide biobank containing both interview data and biological material. The overall objective was among children born in Denmark to collect data for studies of exposures to early childhood and their long lasting impact on health and disease. The DNBC leaves unique possibilities for study of time-dependent changes in disease incidence.

The aims of the present study were to estimate and validate the national age-specific disease burden of OM including ventilation tube insertion.

## Methods

The study is a registry-based national cohort study of children enrolled in the DNBC from 1996–2003. Sixty percent of Danish speaking women were invited to participate at the first visit to their general practitioners during pregnancy. Approximately 35% of all pregnant women during the study period participated (101,042 women). The women provided their written informed consent to participate in the DNBC. This was approved by Ethics Committee.

The DNBC data collection was based on telephone interviews performed by trained interviewers. In this study we used interviews 3, 4, and 5 that were carried out when the children were 6 and 18 months and 7 years old. Among the several hundreds of questions in the DNBC a few concerned OM. There were no specific questions about the exact types of OM (AOM, OME and CSOM) because parents often have difficulties in differing between these diagnoses. Therefore in this study OM denotes all types of OM. The women who participated in the DNBC were slightly older (60% were 30 years or older) than those who were lost to follow-up. The women who were lost were generally more overweight before they became pregnant, had lower occupational status and were heavier smokers [12]. Further details of the DNBC has been described elsewhere [11]. Excluded were children with cleft palate or Downs syndrome (n = 260).

We tested internal and external validity of OM related questions in the DNBC. The internal validity of OM information in the DNBC was tested by comparing answers to the question ‘Has your child ever had middle ear infection?’ in the 6-month, 18-month and 7-year reportings and by comparing answers to the question ‘How many times has he/she had a middle ear infection?’ in the 6 months- and 18-months interviews. The external validity was tested by comparing responses to the question ‘Has your child ever had ventilation tube inserted’ from the 7-year interview with information from national registries of insertion of ventilation tubes in private ear-nose-throat clinics and public hospitals. For Danish citizens all treatments are paid for by the state. All treatments are registered on a person identifiable basis: for private clinics in the National Health Insurance Service Registry (www.ssi.dk/Sundhedsdataogit/Registre/Sygesikringsregister) and for public hospitals in the National Patient Registry (www.ssi.dk/Sundhedsdataogit/Registre/Landspatientregistret), (procedure codes 3009 and 3109 and ICD10 procedure code KDCA20 cover all ventilation tube insertion).

The study was approved by The Danish Data Protection Agency and the DNBC was approved by Ethics Committee.

## Statistical Analyses

All analyses were performed using SAS Ver. 9.2.

The cumulative risk of OM until 18 months of age was estimated by Kaplan Meier plots with 95% confidence intervals (CI) based on the 6- and 18-month interview. The 7-year interview did not include a question of the exact age at debut. To estimate the cumulative incidence at 7 years, the binomial proportions from a SAS Freq procedure was used, where only persons having answered the equivalent of ‘yes’ or ‘no’ in the 7-year interview was included (in total 52,755 persons).

Age-specific incidence for children aged 0–18 months was based on the 6- and 18-months interviews. For each period this was calculated by dividing number of OM cases for the specific time period by the number of child months at risk. The responses “unknown” or “unaware” (n = 1923) to OM questions were excluded. Rates of recurrent OM episodes by gender were based on the 18-months interviews containing detailed questions about the number of OM episodes. The association between early debut of OM and recurrent episodes was determined using all interviews if the child had had four or more episodes by the age of seven years and the age at first episode was determined through the 6- or 18-months interviews.

When calculating internal validity regarding number of episodes, the participants were not allowed to indicate a lower number of OM episodes in the 18-months interview than in the 6-months interview or to indicate OM episodes in the 6-months and not having any OM episodes in the 18-months interview. If any mismatch in the interviews, information from the earliest interview was used. All participants replying to questions on OM regardless of whether the child had had an OM episode or not were included.

External validity of questionnaire information on ventilation tube insertions was calculated using information from the 7-year interview and the estimation of sensitivity, specificity, positive predictive value, negative predictive value, and Kappa value was done against national registry information as gold standard.

## Results

Of the total cohort of 101,042 women, 95,095 women (94.1%) carried their pregnancies to term. Of full term pregnancies 70,294 (73.9%) women replied to the 6-month’s interview; 66,712 (70.2%) to the 18-month’s interview, and 53,888 (56.7%) to the 7-year interview. 86,441 (90.9%) replied to at least one of the three interviews and 32,199 (33.9%) replied to all. Table 1 presents participants and their demographic characteristics.

**Table 1:** Baseline characteristics of 54,772 children in the Danish National Birth Cohort 1996–2005, Denmark. Parents who responded both 6- and 18-months interview.

Characteristic	+ OM n (%)	− OM n (%)	OR adjusted*****(95% CI)	p-value (OR)
**Sex**				<0.0001
Boy	12,030 (54.6)	15,834 (48.4)	1.3 (1.2–1.3)	
Girl	10,025 (45.5)	16,883 (51.6)	1 (Reference)	
**Breastfeeding (months)**				<0.0001
Never	97 (0.4)	132 (0.4)	1.2 (0.9–1.6)	
0−<2	2,591 (11.8)	3,121 (9.5)	1.3 (1.3–1.4)	
2−<4	2,020 (9.2)	2,595 (7.9)	1.2 (1.2–1.3)	
4−<6	2,814 (12.8)	3,627 (11.1)	1.2 (1.2–1.3)	
>6	14,520 (65.9)	23,231 (71.0)	1 (Reference)	
**Passive smoking**				<0.0001
Never	17,949 (40.1)	26,813 (59.9)	1 (Reference)	
At 6- and 18- months interview*	1,796 (39.6)	2,471 (60.4)	0.9 (0.9–1.0)	
At 6-months interview**	670 (46.7)	766 (53.3)	1.3 (1.1–1.4)	
At 18-months interview***	1,625 (40.6)	2,380 (59.4)	1.0 (0.9–1.1)	
**Daycare**				<0.0001
Never	1,886 (8.6)	4,197 (12.8)	1 (Reference)	
At 18-months interview****	4 (−)	6 (−)	2,7 (0.2–3.2)	
At 6-months interview**	2,782 (12.6)	3,706 (11.3)	1.6 (1.4–1.7)	
At 18-months interview***	17,383 (78.2)	24,805 (75.8)	1.6 (1.5–1.7)	

*Child exposed to passive smoking in both 6- and 18-months interview.

**Child exposed to passive smoking in the 6 months interview only/introduction age to daycare.

***Child exposed to passive smoking the 18-months interview only/introduction age to daycare.

****Child exposed to daycare in the 18-months interview, but information about first introduction is missing.

*****OR adjusted for variables included in table 1 (breastfeeding/smoking/daycare).

**Figure 1:**
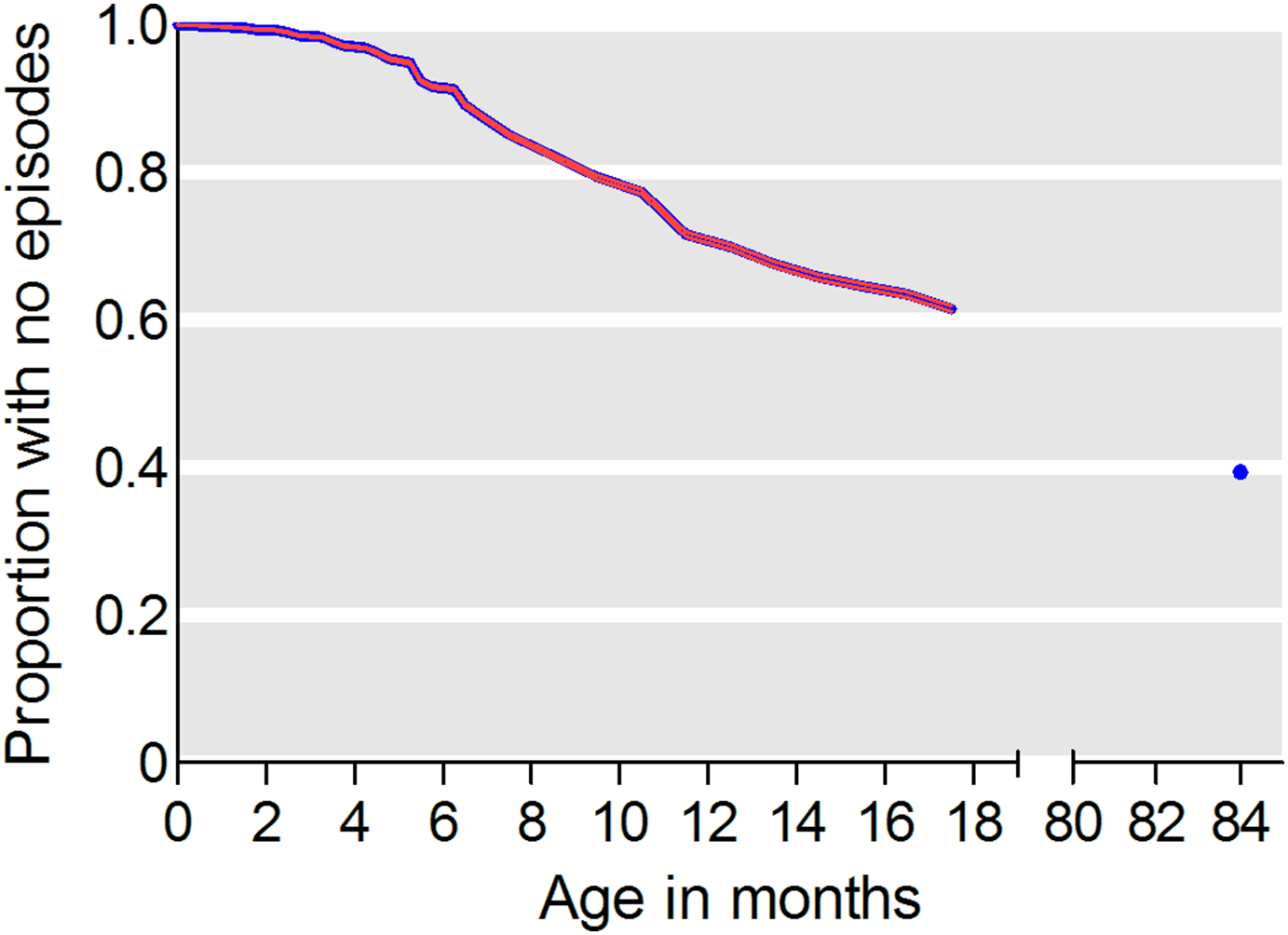
Cumulative incidence of OM 0–7 years of age. Kaplan Meier survival curve with 95% confidence intervals (red line and dot) showing the cumulative incidence of otitis media (OM) in 81,374 children aged 0 months–7 years of age.

**Figure 2:**
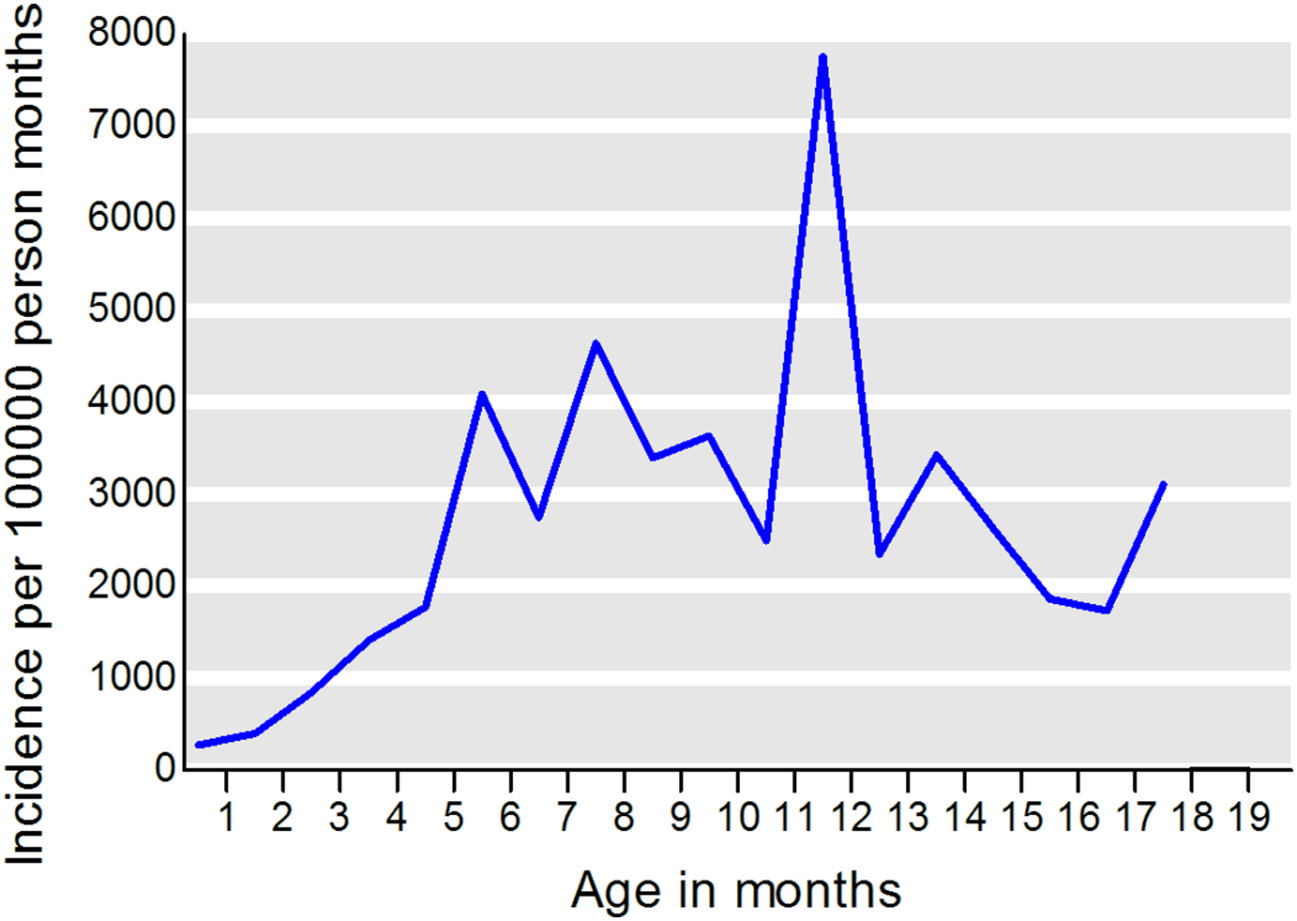
Risk of OM in the first 18 months of life. Risk of OM in the first 18 months of life (blue line) among 86,044 children in the Danish National Birth Cohort. Based on parents who responded the 6-months, the 18-months or the 7-year interview.

In total 31,982 mothers reported their child having had OM at any time during interviews corresponding to a cumulative incidence of 60.6% at age 7 years (95% CI 60.2–61.0). Fig. 1 shows cumulative incidence of OM. Table 2 shows frequency of OM at each interview. The median age at first OM episode was 10 months (25–75% quartiles 6–12 months); 10 months (25–75% quartiles 7–13 months) for girls; and 9 months (25–75% quartiles 6–12 months) for boys (p<0.0001). At 18 months of age 38.8% of children had had at least one episode of OM, and in the period from 18 months to 7 years of age an additional 21.5% of children reported new first OM episodes. Fig. 2 shows the age-specific incidence of OM before 18 months of age. The risk peaked around one year of age and was lowest before five months of age.

**Table 2:** Frequency of otitis media (OM) among children aged 0–7 years of age in the Danish National Birth Cohort, 1996–2005, Denmark.

	OM/participants	Frequency	CumulativeFrequency	95% CI
At 6-months interview	3,711/70,294	16.2%**	5.3%	5.1–5.5
At 18-months interview	25,896/66,712	44.3%**	38.8%	38.7–39.4
At 7-year interview	31,982/52,755*	39.5%**	60.6%	60.2–61.0

*missing 1133 (lack of responses/other responses than “yes”/“no”).

**Frequency at specific interview in time period from previous interview.

Four or more episodes of OM were reported by 0.3%, 13.6% and 23.9% of children at the 6 months, 18 months, and 7 years interviews, respectively, or 39,5% from birth to 7 years of age. Of those who reported OM debut between 0–6 months of age, 64% (2482/3881) (3881 are those who replied “yes” to having an OM episode before 6 months of age. Of those who indicated an OM episode before 6 months of age, 2482 had more than four episodes of OM) later reported having had ≥4 OM episodes and for those with OM debut between 7–18 months and 19 months-7 years these proportions were 48.2% (4998/10378) and 28.7% (4996/17344), respectively (p<0.0001). Table 3 shows the number of OM episodes reported at the 18 months’ interviews. Of those with OM a total of 57.3% children had 2 or more episodes before 18 months of age with boys having significantly more than girls (p<0.0001) regardless of the number of episodes. At 7 years of age 13,988 (26.1%) [95% CI: 25.7%–26.4%] had had a ventilation tube insertion at least once corresponding to 43.1 per 1000 per year.

**Table 3:** Number of episodes of otitis media (OM) at the 18 months’ interview in 32,459 girls and 33,762 boys in The Danish National Birth Cohort, 1996–2005 Denmark.

Number ofOM episodes	Boys (%)n = 33,762	Girls (%)n = 32,459	Total (%)	95% CI*	p-value
0	58.2	63.9	40,383 (61%)	60.6–61.4	<0.0001
1	16.9	16.5	11,059 (16.7)	16.4–17.0	<0.0001
2–3	9.2	8.2	5,749 (8.7)	8.5–8.9	<0.0001
4–6	10.9	8.3	6,344 (9.6)	9.4–9.8	<0.0001
7–10	3.1	2.1	1,728 (2.6)	2.5–2.7	<0.0001
>10	1.8	1.1	958 (1.5)	1.4–1.5	<0.0001

*Confidence interval.

### Questionnaire validation

Table 4 shows the distribution between parental reporting of ventilation tube insertions and information in national registries. Overall, there was high agreement between the two with kappa value 0.941 (95% CI 0.938–0.944), sensitivity 96.4%, specificity 98.2%, positive predictive value 94.8% and negative predictive value 98.8% of answers in the questionnaires.

**Table 4:** 2×2 table of tube insertion information in 53,696 (missing 192) children from the Danish National Birth Cohort, 1996–2005.

		Questionnaire information	
		Yes	No
**Register information**	Yes	13,262 (24.7%, 95% CI 24.3 25.1%)	493 (0.9%, 95% CI 0.8–1.0%)
	No	726 (1.4%, 95% CI 1.3–1.5%)	39,215 (73%, 95% CI 72.7–73.4%)

Information in the 7-year interview in the Danish National Birth Cohort and national Danish register information (National Patient Registry or National Health Insurance Service Registry).

The agreement between parents’ answers to the question “*Has he/she ever had otitis media?*” at the 6 and 18 months’ interviews was 98.8%, and 95.3% at the 18 months and 7 years interviews, respectively. Only 59 (0.1%) of 54,958 mothers gave contradictory answers at the 6-month and 18-months interviews to the question *“How many episodes of otitis media has he/she had?”.*

## Discussion

This is the largest birth cohort study of OM incidence in the world with 95,095 live-born children followed from 1996. OM is still a highly frequent disease among children in Denmark with a cumulative incidence of 60.6% before 7 years of age and 39.5% of those with OM reported to have had ≥4 OM episodes before 7 years of age and 26% of the children had been treated with ventilation tubes at least once. Both external and internal validity of the questionnaire information in the DNBC was high.

Twenty to 35 years ago epidemiologic studies from western countries showed high frequencies of OM in childhood. In Finland, 1982, 76% of children aged 6–11 months had had AOM with 50% and 75%, respectively, having experienced at least one OM episode before the ages of 3 and 10 years [13]. In Sweden, 1985, 60% of boys and 55% of girls had experienced AOM before 5 years of age [14], and in 1990 Schappert found that OM was the most frequent diagnosis in general practice in the USA among 24.5 million visits for children under 15 years of age [15].

In Denmark in 1975 and 1979, 50%, 70% and 75% of children at the end of their 3rd, 5th and 9th living year, respectively, had experienced at least one AOM episode [5], and 41% of children with an average age of 3½ years (n = 494) had experienced ≥ one OM with 26% having had > 1 episode of OM (mean: 3.8 OM episodes). 51% and 75% of those with OM had had their first episode before their 1st and 2nd years of age [4]. In 1981 among 387 children screened by 10 tympanometries in one year, Lous et al. found that at 7 years of age 26% had OME and 43% had tubal dysfunction with a C-tympanogram [16]. Although direct comparisons are difficult, our figures with cumulative incidences of 39% at 1½ years of age and 61% at 7 years of age are not markedly different but 57% had experienced ≥ two OM episodes before 18 months of age, which is more than twice as many children than reported in 3½ year-olds in 1979 [4]. There are methodological differences as the former studies all included smaller, and geographically selected populations while our study was a nationwide population-based cohort followed for 7 years. Our study was an interview based questionnaire without exact knowledge of specific OM types. Yet, it is surprising that the incidence does not seem to have changed over the last 25–30 years. However, we can only compare our study with other studies based on a similar design with questionnaires from time equal periods. At 18 months of age we found a cumulative incidence on 38.8%, for the Norwegian cohort this was 30.4% [1], and for a cohort in the Netherlands is was 39.5% (cumlative incidence from 6–24 months) [17].

In the Danish population passive smoking has decreased and 90% of children in the DNBC were not exposed to daily passive smoking, whereas in 1991, 50% of all Danish children were exposed to passive daily smoking [18]. Maternity leave has been extended and therefore in general children start at an older age in childcare centres [19]. In the cohort 83% started day-care later than 6 months of age. However, in Denmark today more children attend childcare centres than 25–30 years ago [20] which tends to oppose the decrease in other risk factors for OM. In a study by Vinther et al. from 1979 [4], 70% of the children attended day-care. For our study this was 88% of the children. The Danish Board of Health has produced guidelines for breastfeeding and 80% of our children were breastfed for ≥4 months In Denmark in 1971, 1976–77, 1978 and 1991, respectively, 27%, 32%, 47% and 73% of children were breastfed at 3 months of age [21]. Thus, the net changes of the risk factors for OM are of limited importance or factors, known or unknown, increasing or reducing the risk of OM may balance each other.

Our study shows that OM in Denmark still constitutes a significant burden of disease.

The increased risk at exact age 12 months is most likely due to parental recall bias as parents might easier remember a round figure of e.g. ‘1 year’ than a specific number of months e.g 11 months or 13 months. In Denmark day-care most often begins at 1-year of age increasing the risk of infections. OM is considered a disease with debut in very early childhood but in this study 22.1% of new OM cases occurred between 18 months and 7 years of age.

We found as others [4] an association between early onset of OM before 6 months of age and recurrent episodes of OM. Recurrent episodes might be caused by a less functional Eustachian tube and an immature immune system.

Passive smoking between 0–6 months of age, introduction to daycare and male gender increased the risk of having OM. However, we also found that there was no difference between having and never having been breastfed which is in contrasts to many other studies [22]. The never breastfed group in our study was, however, very small.

We found until now the highest rate in the world of ventilation tube insertion [23], [24] with 26% of children having had at least one event of tube insertion before 7 years of age corresponding to 43.1 per 1,000 per year. In 2000 in Maryland, USA, 6.8% of children by age of 3 had tubes inserted [25]; in Finland and Norway children younger than 17 years of age experience rates of ventilation tube insertion of 51.3 and 43.2 per 10,000 children (0.5 and 0.4%), respectively, with peak ages for surgery at 3 and 6 years of age [26]. In 2004, in the UK tubulation rates were 2 per 1,000 (0.2%) per year in children younger than 15 years, and in the Netherlands 20 per 1,000 (2%) per year in children younger than 12 years [27]. Thus, there is a large variability in ventilation tube treatment in Western countries. Treatment of OM with ventilation tubes in Denmark is most often performed after 3 months’ observation (‘watchful waiting’) [28]. The high rates of ventilation tubes in the Netherlands and Denmark might be explained by a more restrictive antibiotic policy, cultural or unknown differences compared to other western countries [27], [29].

Several countries (e.g. Sweden and the US) have during recent years introduced national guidelines for treatment of the different types of OM [23], [24]. At present Danish guidelines are under preparation but still not existing.

The main strength of this study is the large size and representativeness of the cohort, comprising 95,095 unselected children with information of exposures and outcomes [11]. We used Danish National register information. There was short time between the early interviews, minimizing recall bias but our study may be subject to recall bias and diagnostic bias especially for the 7 years interview. However, we found a 95,3% agreement in positive OM answers at the 18 months and 7 years interviews indicating acceptable validity with a relatively low risk of recall bias also in the 7 years interviews. Also, the DNBC contained several questions concerning diseases often associated with upper respiratory tract infections (URI) in general such as cough, asthma, rhinitis and pneumonia. It is therefore most likely that the OM diagnosis at the 7 years interview is not mistaken as URI and that this tends to lower the risk of diagnostic bias.

OM questions were only a very small part of the total DNBC interviews and therefore it is unlikely that parents without OM children were less interested in participating in the interviews. Furthermore, as healthcare is free to all citizens in Denmark, the risk of socio-economic selection bias in OM children and children without OM in the cohort is reduced.

The inclusion to the DNBC cohort took place from 1996–2003 before the pneumococcal conjugate vaccine 7-valent (PCV7) vaccine was introduced after the 30 April 2006, without any catch-up programme. This can lead to that more children in our cohort suffered from OM, than if they had been given the vaccine. However a Cochrane review concludes that the PCV7 has only marginal effects on the overall OM prevalence [30]. In 2010 PCV13 was introduced in Denmark. Neither have the children in our cohort been given this vaccine, which can also cause that more children in the DNBC had OM. A study from 2014 from US showed that there was an overall decrease in OM in children younger than 2 years from 2010–2011 in children in the US probably due to the introduction of the PCV13 [8].

The vaccinations to children in our cohort were the diphtheria-tetanus-pertussis-polio/Hib and the measles-mumbs-rubella vaccine. Diphtheria-tetanus has been given since 1950. Polio from 1955. Pertussis from 1961. Measles-mumbs-rubella from 1987. Hib from 1993. According to Statens Serum Insitute 95% of Danish children follow the Danish Childhood Vaccination Programme.

Another limitation of this study is information is based on parental interviews liable to erroneous recall and not on physician reported clinical data. However, both external and internal validity of questionnaire information was high. Correspondingly, studies from the DNBC found high agreement between information given by parents and different clinical diagnoses [31], as well as low rates of bias [32]. Yet, we could only validate the questionnaire information regarding tube insertions and not physician-diagnosed OM, as this information is not available from Danish registers. Detailed information about clinical manifestations of OM (AOM, OME, or CSOM) would have been warranted. Furthermore, 40% of invited GPs and 1/3 of addressed mothers actually participated in the DNBC [11]. The socioeconomic pattern of the participants in the DNBC has recently found women with no jobs, no education other than compulsory school and women in the lowest income group to be underrepresented by 62%, 43% and 22%, respectively, but with higher risk behaviour [12], [33], indicating that non-participants are more at risk of several diseases. This is a well-known bias in large population-based studies. Also, only Danish speaking women were invited to participate in the cohort, which may have increased risk of bias [11]. It is difficult to estimate whether the participation rate by GPs lead to bias but the low participation rate among the lowest income group is expected to lead to underestimation of the frequency of OM. Surprisingly, Lous et al. recently found that children of social marginalized mothers in Denmark pay fewer visits at ENT physicians, and have less commonly insertion of ventilations tubes done compared with children of non-marginalised mothers [34].

Erroneous recall may in particular have occurred in the 7-year interview as the parents were asked to report OM for the whole period of 7 years. However, the first interviews at 6 and 18 months included more than 2/3 of first OM episodes, and the relatively shorter interval between these interviews makes estimation of the precise age at debut reliable.

Previous studies from the USA and Denmark have found that parents’ reporting OM in their children correlate well with medical files [31], [35]. However comparison with former studies is difficult as study designs are different from ours and studies that validate parents’ answers on OM with a long follow up period do not exist. Also, it has been claimed that as parents’ reporting OM in interviews is often based on the child having fever, crying or restless behaviour, this may cause misclassification since these symptoms are not specific for OM and therefore cause overestimation of OM results [36]. Otoscopy is complicated in toddlers with narrow ear canals often obstructed by earwax. Optimally, the tympanic membrane should be viewed with a microscope, which is generally not used by general practitioners. Even among ENT specialists OM can be difficult to diagnose [37]. In all these sources of bias may tend to overestimate the rate of OM, but other similar studies are subject to the same bias.

## Conclusion

In conclusion, this nation-wide birth cohort study of children aged 0–7 years showed a cumulative risk of OM of 60.6% before 7 years of age that surprisingly has not changed in the past 35 years. Early onset of OM is associated with higher risk of recurrent OM, the risk of OM is highest between 7–19 months of age, and 26.1% of children in Denmark have been treated with ventilation tube insertion before 7 years of age. Compared with information in Danish high-quality registers, the validity of questionnaire information in this study was high.
